# Supplementary material for: Sequence and structure comparison of ATP synthase F0 subunits 6 and 8 in notothenioid fish
Source: PLoS One. 2021 Oct 6;16(10):e0245822. doi: 10.1371/journal.pone.0245822 (PMC8494342; doi:10.1371/journal.pone.0245822)
Supplement: S1 Fig — (DOCX) [file pone.0245822.s001.docx]

Supplementary Figure 1. A pictographic representation of the relatedness of ‘ATP6 protein’ sequence for notothenioids to other species using NJ-phylogenetic tree (Clustal omega[35]) analysed by taking alignment data that shows similarity in the amino acid composition of the protein for different vertebrate species (pictures source: wikipedia.com, human skull: bonesclones.com, naked mole rat: wikiwand.com, *E. maclovinus*: scanndposters.com).

In the study we have aligned the nucleotide and protein sequences for ATP6. The choice of different vertebrate species as outgroups apart from the notothenioids (Supplementary Fig 1.) for the protein allow us to highlight changes in the amino acid sequences that may be specific to the notothenioid fish and those changes that may be species specific.
