# Supplementary material for: Sequence and structure comparison of ATP synthase F0 subunits 6 and 8 in notothenioid fish
Source: PLoS One. 2021 Oct 6;16(10):e0245822. doi: 10.1371/journal.pone.0245822 (PMC8494342; doi:10.1371/journal.pone.0245822)
Supplement: S2 Fig — (DOCX) [file pone.0245822.s002.docx]

Supplementary Figure 2 . Protein structure evaluations of ATP6 for fish species *C. aceratus*, *C. gunnari*, C*. rastrospinosus*, *E. maclovinus*, *N. corriceps*, *T. bernacchii*, *D. rerio* and *N. furzeri* (A-I) using SAVES v6.0 (https://saves.mbi.ucla.edu/), using ERRAT[41], PROCHECK[42,43] and ProSA-web[44].

The structures are predicted models with 93-95% overall score for the model (ERRAT, the method provides an unbiased and statistically sound tool for identifying incorrectly built regions in protein models). The Ramachandran plot on average showed allowed regions from >85 %. An acceptable high-quality model will have allowed regions >90%, and this low percentage can be either attributed to either high active side residues that sometimes are found in disallowed regions or low resolution of the model that can have disallowed conformations (Supplementary Fig 2.).
