# Supplementary material for: Sequence and structure comparison of ATP synthase F0 subunits 6 and 8 in notothenioid fish
Source: PLoS One. 2021 Oct 6;16(10):e0245822. doi: 10.1371/journal.pone.0245822 (PMC8494342; doi:10.1371/journal.pone.0245822)
Supplement: Attachment — Submitted filename: ResponseToReview.docx [file pone.0245822.s003.docx]

Dear Editor,

Thank you for giving us the opportunity to submit a revised draft of our manuscript

*“Sequence and structure comparison of ATP synthase F0 subunits 6 and 8 in notothenioid fish”* to *PlosOne*. We appreciate the time and effort that you and the reviewers have dedicated to providing your valuable feedback on our manuscript. We have endeavored to incorporate changes and address the suggestions of the reviewers. We have highlighted the changes within the manuscript.

Here is a point-by-point response to the reviewers’ comments and concerns.

**Comments from Reviewer 1**

- **Comment 1**: “*The study is original and with a high potential interest, but not clear conclusions are stated in the Abstract and/or Discussion sections.*”

**Response**: Thank you for showing interest in our work. We agree with this comment, therefore, we have made changes in the abstract and discussion with explanation of what we have found and are seeing where in the revised manuscript this change can be found.

- **Comment 2**: “conducting some physiological validation if it is needed”

**Response:** We have performed western blot for the protein ATP6 on whole cell fractions of liver samples isolated from four notothenioid species, *N. rossii* and *T. bernacchii* (the red-blooded species) and *C. rastrospinosus* and *C. gunnari* (the icefish). The ATP6 protein was seen significantly lowered for icefish *C. gunnari* in comparison to the other species. This might be correlated with the changes observed for alternate start codons for the other species and specific amino acid substitution. GAPDH was used as a loading control for the samples. This is very preliminary data which we hope is the start of a comprehensive experimental study to look more closely at these proteins in the ice-fish. Physiological validation will depend to some great extent upon availability of tissues from these species (which is difficult to obtain). Further in-vitro work that is planned would be beyond the scope of what is presented i.

Figure 1. Liver tissues from *N. rossii*, *T. bernacchii*, *C. rastrospinosus* and *C. gunnari* whole cell lysate separated on SDS-PAGE and analysed by Western blotting using antibody against 25-kD subunit of ATP synthase, ATP6 using GAPDH as a loading control.

**Comments from Reviewer 2**

- **Comment 1:** “*The article opens with a relatively unclear goal: “shed light on the molecular evolution of these proteins in vertebrate species,” meant ATP synthase F0 subunit components coded by mtATP6 and mtATP8. This goal doesn’t seem to correspond with the manuscript title so much.*”

**Response:** We agree with this and have incorporated your suggestion throughout the

manuscript by making the statement clearer to correspond better with the title of the paper eg. Introduction lines 105-114.

- **Comment 2:** “*An unclear statement: “The initial difference we see is that red-blooded species N. coriiceps, E. maclovinus, T. bernacchii, and the two icefish, C. rastrospinosus that are devoid of haemoglobin but have myoglobin and C. aceratus species with a nearly identical gene to that of C. rastrospinosus, have an additional 12 nucleotides at the N-terminal. The only exception to this is the icefish C. gunnar*i”

**Response:** We agree with this and have incorporated your suggestion throughout the

manuscript and can be seen particularly in changes from the very start of the discussion.

- **Comment 3:** “*Relatively short, but not so clear subchapter: “Overlap of ATP8 and ATP6 genes.*”

**Response:** We agree with this and have incorporated your suggestion and added more relevant explanation for the subheading “overlap of ATP8 and ATP6 genes”. This change can be found under the heading ‘overlap of ATP8 and ATP6’, lines from 340.

- **Comment 3:** “*Relatively long, but not so clear subchapter: “Protein Alignment and Structural changes in ATP6”. Conclusion section is missing.*”

**Response:** We have incorporated the changes in the manuscript from line 339.

- **Comment 4:** “*Many of their results are not even discussed. So, I recommend the authors to articulate clearly their research goal first, then select carefully the best Materials and Methods allowing them to reach their goal, then comment all their results properly in Discussion and provide the reader with a general Conclusion at the end*.”

**Response:** We have altered the manuscript in response to this comment and think it is now improved we have also added a conclusion.

- **Comment 5:** “*Abstract, line 27 “The Channichthyidae family “; Introduction, line 56 “Within the Nototheniidae family the subfamily Channichthyinae” and so on. The authors should select the taxonomic system they consider correct and stick to it in the whole article (I suggest following Near et al 2018). The current text is very confusing when authors are using multiple systems in parallel, without any explanation, and they aren’t even testing any hypotheses intended to correct the taxonomic system in use.”*

**Response:** The manuscript has been altered to be consistent in its taxonomic references, specifically, it now refers to Channichthyidae as a family within the Notothenioid suborder.

- **Comment 6:** “*Introduction, lines 62,63 “sixteen species of the Channichthyinae subfamily” cited sources 1997, 2003. The Channichthyidae family (or Channichthyinae subfamily) covers 16-23 described species in 11 genera. The views of different researchers on the species composition of the taxon differ a lot, especially within the nominotypical genus Channichthys (https://link.springer.com/article/10.1134%2FS0032945219060079; https://www.zin.ru/journals/trudyzin/doc/vol_323_4/TZ_323_4_Nikolaeva.pdf). So, I suggest avoiding such explicit controversial statement, because the authors aren’t even testing any hypotheses intended to resolve the situation.*”

**Response:** The authors have removed reference to a total number of Channichthyidae species, and simply state that only six species of Channichthyidae have been reported as lacking myoglobin within their cardiac muscle.

- **Comment 7:** “*In the final paragraph of Introduction, the authors list the species compared and they provide the Latin name twice. I suggest providing the full scientific name just once in this paragraph, but properly instead, e.g., Champsocephalus gunnari Lönnberg, 1905; and short in the rest of their text, e.g., Ch. gunnari. Furthermore, I suggest adding the family/subfamily information there, because the authors describe the differences in notothenioid thermo-adaptation in family/subfamily levels earlier in the Introduction.*”

**Response:** The authors have corrected the final paragraph to include just one usage of the Latin name of each species considered in the manuscript, and specify family information of the Channichthyidae species under consideration.

- **Comment 8:** “*The information is repeated in the Methods, Results, and Fig 5 caption: “Sequence logos were created using the webserver WebLogo using 4000 vertebrate protein sequences for the protein ATP6 (http://weblogo.threeplusone.com/).” Unfortunately, the authors don’t provide any detail about the 4000 sequences selection criteria. The authors should avoid such redundant repeating of information, but they should provide all relevant information allowing a verification of their result, in the Method section. Anyway, I didn’t notice these results mentioned in the Discussion.”*

**Response:** The analysis was repeated and a list of 5947 sequences were aligned and used for generating the logo. The information is added in methodology, results and discussion.

- **Comment 9:** “*Only the protein based dendrogram (Fig. 3) is mentioned in the text (Method and Result sections) but not the DNA based dendrogram (Fig. 4). The protein based dendrogram (Fig. 3) suggests that zebrafish and killifish are phylogenetically related to a lizard, perhaps even more than to notothenioids, which is obviously not true. Fortunately, it’s well established that lizard is not a teleost. Phylogenetic analysis anticipates selection neutrality of the sequence analyzed, which clearly is not the case in the notothenioid ATP synthase F0 subunits. The distance-based NJ dendrograms thus don’t reconstruct the phylogeny of taxa analyzed, but they are reflecting the unique evolutionary history of these sequences under selection in notothenioids instead. It is interesting that the ATP synthase F0 subunits protein and DNA based dendrograms within the Channichthyidae family correspond with each other and the well-established RADseq based phylogenetic analysis of this clade (Near et al 2018), but it doesn’t apply on the whole notothenioid clade anymore. The sentences in Result section describe this insufficiently. Nonetheless, neither these analyses nor phylogenies are mentioned in the Discussion (and Introduction) at all, which leaves me wondering why the authors performed such analyses. The visualization of these dendrograms is also quite confusing and it deserves more polishing in iTOL so the protein based dendrogram (Fig. 3) is visualized equivalently to the DNA based dendrogram (Fig. 4) at least. These dendrograms could be also rooted, because we know the phylogeny in advance.*”

**Response**: The phylogenetic analyses didn’t seem necessary or fit with the current study and have therefore been removed.

- **Comment 10 :** “*Table 1 and Table 2 are sharing 5 rows, while Table 1 contains 10 rows in total. So, I think that merging these two tables would simplify the reporting and it would be easier to follow.*”

**Response:** We agree with the comment and have combined Table 1 and Table 2 together as Table 1.

**Comments from Reviewer 3**

- **Comment 1:** “*In the introduction it is mentioned that this work is carried out through the use of sequence analysis and secondary structures, however, the analysis carried out was based on tertiary structure, no secondary structure was determined in this work (see: 123-125)*”

**Response:** We agree with this and have changed the line.

- **Comment 2:** “*The I-TASSER tool was used for the prediction of tertiary structures. However, in this work the quality of the modelled structures is not verified (Ramachandran plot, Z-score, etc.), which raises questions about the results. This is a very critical aspect*”

**Response:** We agree with this and have incorporated your suggestion throughout the

manuscript. The validations are submitted as supplementary files. The structures are predicted models with 93-95% overall score for the model (ERRAT, the method provides an unbiased and statistically sound tool for identifying incorrectly built regions in protein models). The Ramachandran plot on average showed allowed regions from >85 %. An acceptable high-quality model will have allowed regions >90%, and this low percentage can be either attributed to either high active side residues that sometimes are found in disallowed regions or low resolution of the model that can have disallowed conformations.

- **Comment 3:** “*Structural comparisons are made at work, however, the way to proceed is not correct. For this analysis, structural protein alignments (superimposed structure) are performed where measurements such as the TM-score and RMSD are determined, which allow establishing functional relationships even when the sequence similarity is low.* *For this analysis there are different programs, an excellent one is "RaptorX Structure Alignment Server" to calculate RMSD and TM-score, the software "PyMOL" for RMSD, and the "server TM-score" of the same authors of I-TASSER to calculate the measure with the same name, TM-score.*”

**Response:** Please see methodology section from line 159 where the full structure prediction tools we used are outlined.

- **Comment 4:** - “*I don't understand what he meant by the fact that the Graphs were produced with MATLAB version R2018b (9.5.0). This analysis is not clear. I think it is not recommended to use multiple programs for alignment because they have different algorithms and usually return discrepant results. In case of using them as was done in this work, a more rigorous discussion of the comparative result would be advisable. There is a lack of uniformity in the format of the writing (two types of letters in many parts of the text), for example, in Figure 3. Please, review all text.”*

**Response:** The amino acid properties, molecular weight of amino acids across the sequence, hydrophobicity trend of amino acids, α--helix forming amino acids, average flexibility trend and mutability for the protein ATP6 using ProtScale was used to compare species and the graphs were generated using MATLAB. Multiple programs for alignment haven’t been used. Only Clustal omega has been used for comparing sequences.

Response to comments that we disagree with

**Comments from Reviewer 1**

- **Comment 1:** “*In previous studies in fish model species and farmed fish (including salmonids and non salmonids), the specific regulation of enzyme subunits of the mitochondrial respiratory chain has been addressed in response to changes in food availability and other stressors. These and other physiological studies in other vertebrates should be considered to improve the Discussion section*.”

**Response:** Though we definitely understand the importance of food and other stressors we feel that this subject area is rather far removed from that of our study to be included at this point.

- **Comment 2:** “*The study is focused on ATP6 and ATP8, but why not other enzyme subunits of the respiratory chain encoded by the mitochondrial or nuclear DNA. A more ambitious study, addressing changes in enzyme subunits of the five enzyme complexes of the mitochondrial respiratory chain should be considered.*”

**Response:** Thank you for this suggestion. It would have been interesting to explore this aspect. Our main aim was to study complex V in these species due to tight coupling of icefish mitochondria and any changes in this subunit could directly have an impact on physiological outputs (as also mentioned in our introduction). Only these two subunits of this complex, ATP6 and ATP8 are encoded by the mitochondrial DNA (mtDNA). The mtDNA is known to evolve faster and is ideal for evolutionary studies. Also, in the case of our study, the reviewer’s suggestion seems unrealistic because the sequences of the other proteins are not sequenced for the notothenioid species.

**Comments from Reviewer 2**

- **Comment 1:** “*The authors didn’t generate their own data and they are using published data instead. The authors didn’t develop any new method of data analysis and their results were generated often by easy-to-use web-based software. Majority of the analyses were done on just 13 model vertebrate species*.”

Response: Many bioinformatics studies utilise and make sense of already available biological data that has not been analysed in this manner previously. Much of the data that is gathered in omics projects is submitted and not compared with other datasets that can reveal novel biology. We hope the reviewer will accept that data are only useful after thorough analysis and not just as accession numbers in databases. The analyses were done on 13 very different vertebrate species which makes the study unique.

• **Comment 2:** “*The authors don’t explain so clearly the reason for their model organism selection, which is questionable. The authors picked 8 teleosts, 1 lizard, 4 mammals (but not human); no cartilaginous fish, no lamprey, no hagfish; no tunicate and/or lancelet as an outgroup.*”

**Response:** In response the human sequences for the protein have been added to the MSA. We chose this set of since we wished to evaluate our analyses with established sequences, while also keeping in mind the diversity of including different vertebrate groups. We have included statements to clarify our choices in the methodology and discussion.

In addition to the above comments, all spelling and grammatical errors pointed out by the reviewers have been corrected. We look forward to hearing from you in due time regarding our submission and hope that you will be pleased as we are with the revised version.

Sincerely,

Lisa Chakrabarti B.Sc (Hons)., D.Phil (Oxon)
